# Aspirin enhances cisplatin sensitivity of resistant non-small cell lung carcinoma stem-like cells by targeting mTOR-Akt axis to repress migration

**DOI:** 10.1038/s41598-019-53134-0

**Published:** 2019-11-15

**Authors:** Poulami Khan, Apoorva Bhattacharya, Debomita Sengupta, Shruti Banerjee, Arghya Adhikary, Tanya Das

**Affiliations:** 1 Division of Molecular Medicine, Bose Institute, P-1/12, CIT Scheme VII M, Kolkata, 700054 India; 2 Present Address: Centre for Research in Nanoscience and Nanotechnology, University of Calcutta, JD-2, Sector III, Salt Lake, Kolkata, 700098 West Bengal India

**Keywords:** Cancer stem cells, Metastasis

## Abstract

Conventional chemotherapeutic regimens are unable to prevent metastasis of non-small cell lung carcinoma (NSCLC) thereby leaving cancer incurable. Cancer stem cells (CSCs) are considered to be the origin of this therapeutic limitation. In the present study we report that the migration potential of NSCLCs is linked to its CSC content. While cisplatin alone fails to inhibit the migration of CSC-enriched NSCLC spheroids, in a combination with non-steroidal anti inflammatory drug (NSAID) aspirin retards the same. A search for the underlying mechanism revealed that aspirin pre-treatment abrogates p300 binding both at TATA-box and initiator (INR) regions of mTOR promoter of CSCs, thereby impeding RNA polymerase II binding at those sites and repressing mTOR gene transcription. As a consequence of mTOR down-regulation, Akt is deactivated *via* dephosphorylation at Ser^473^ residue thereby activating Gsk3β that in turn causes destabilization of Snail and β-catenin, thus reverting epithelial to mesenchymal transition (EMT). However, alone aspirin fails to hinder migration since it does not inhibit the Integrin/Fak pathway, which is highly activated in NSCLC stem cells. On the other hand, in aspirin pre-treated CSCs, cisplatin stalls migration by hindering the integrin pathway. These results signify the efficacy of aspirin in sensitizing NSCLC stem cells towards the anti-migration effect of cisplatin. Cumulatively, our findings raise the possibility that aspirin might emerge as a promising drug in combinatorial therapy with the existing chemotherapeutic agents that fail to impede migration of NSCLC stem cells otherwise. This may consequently lead to the advancement of remedial outcome for the metastatic NSCLCs.

## Introduction

To inhibit the metastatic property of malignant tumor cells is the most prominent challenge to recent cancer treatments, since the currently available treatment strategies fail to uproot the invasiveness or rather the fatality of the disease. Indeed, over the past 3 decades, the rate of survival of cancer patients has remained mostly unaffected even after conventional chemotherapy^1^. This necessitates for an alternative approach to deal with metastatic human cancers. Growing researches suggests the involvement of a rare subpopulation of tumorigenic cancer cells, named cancer stem cells (CSCs)^2,3^ in metastasis. Clinical and experimental evidences indicate that currently used chemotherapeutic drugs are incapable of affecting human CSCs^4^ that serve as a reservoir for disease recurrence. However, the detailed mechanisms by which these CSCs are involved in initiation and progression of metastasis are still uncertain. This should be immediately detected for developing novel therapeutic approaches to treat metastatic cancers.

Link between PI3K/Akt/mTOR signaling and CSCs are proposed by current literatures. Ser/Thr kinase- protein kinase B (PKB, Akt) is in fact regulates cell survival, proliferation and metabolism^5^. Activation of Akt requires double phosphorylation - one in the activation loop at Thr^308^ and the other in the hydrophobic motif at Ser^473^ ^5^. Phosphoinositide- dependent kinase 1(PDK1) phosphorylates Akt at Thr^308^ ^6^, whereas two component of the PI3 kinase-related super family, mTORC2 (mammalian target of rapamycin complex 2) and DNA-PK (DNA-activated protein kinase) are reported to phosphorylate Akt at Ser^473^ in cellular microenvironment^7^. Moore *et al*. showed that while knockdown or genetic ablation of mTORC2 or Rictor blocks phosphorylation of Ser^473^ i.e. triggered by insulin, absence of raptor has no consequence^5^. In addition to that, purified mTORC2 has been reported to phosphorylate recombinant Akt Ser^473^ directly, but not Thr^308^. However, that mTOR signaling is responsible for maintaining the self-renewal and tumorigenicity of glioblastoma stem-like cells are reported by Sunayama *et al*.^8^. In this regard, activation of Akt by phosphorylation at Ser^473^ residue has been reported to lead to subsequent phosphorylation and inactivation of Akt downstream transcription factor Gsk3β (S9)^9^. In fact, hyper-activated Akt allows phosphorylation of certain important residues of β-catenin by Gsk3β to spot β-catenin for ubiquitination and subsequent proteasomal degradation^10^. According to Zheng *et al*.^11^, Gsk3β phosphorylates Snail, thus ensuring its cytoplasmic translocation for up- regulating E-cadherin expression. Stabilization of Snail protein, by enhanced Akt activity and subsequent Gsk3β phosphorylation, is also observed during EMT in different solid cancer types^12^. However, there is no detail study demonstrating the involvement of this pathway in non-small cell lung carcinoma stem-like cell metastasis.

In this scenario, since the non-steroidal anti-inflammatory drug (NSAID) aspirin has been reported as a potent negative regulator of EMT in NSCLC cells^13^, we aimed at dissecting its role in modulating above-mentioned pathway in non-small cell lung carcinoma stem-like cells, thereby contributing in inhibition of metastasis. However, available standard therapeutic options for metastatic lung cancer include systemic chemotherapy with cisplatin^14^. In breast and cervical cancer, use of cisplatin as chemotherapeutic agent is reported to suppress the growth and proliferation of tumor cells by restraining integrin β5-mediated glycolysis^15^. It is not out of context to mention here that cisplatin resistant cells have been reported to possess Integrin/Fak overexpression^16,17^ that promotes cell migration and proliferation^18^. Several human tumors are also reported to have augmented levels of Fak expression that is in fact, associated with the invasive and metastatic potential of that particular tumor^19,20^. These reports along with the information that mTOR inhibition restores cisplatin sensitivity, prompted us to design a combinatorial therapy of aspirin with cisplatin to effectively inhibit migration of CSCs.

The current study portrays (i) the mechanisms underlying the amplified migration potential of lung cancer stem-like cells (CSC), and (ii) the role of aspirin in modulating the same to sensitize CSCs towards cisplatin. Our search revealed inherent up-regulation of the pro-survival factor Akt in these cells and deactivation of Gsk3β *via* mTOR/Akt axis. As a consequence Snail and β-catenin are stabilized resulting in repression of E-cadherin expression and induction of EMT. In contrast, aspirin inhibits the binding of RNA polymerase II (RNA pol II) onto the TATA box region as well as the INR- region (-1) of mTOR promoter, thereby impeding the activation of Akt pathway and destabilizing the EMT-promoting target genes like β-catenin and snail. Aspirin treatment, though shifted tumor micro-environment in favour of mesenchymal to epithelial transition (MET), failed to suppress the augmented level of Integrin-α2, Integrin-α5, Integrin-β1 and p-Fak, thereby being ineffective in inhibiting migration of CSCs. However, in such anti-migratory micro-environment, cisplatin inhibited Integrin/Fak pathway thereby hindering migration of CSCs. These outcomes propose a promising way of combinatorial approaches of aspirin and cisplatin for achieving CSC-targeted therapy to combat invasive lung cancers better.

## Results

### Migration potential of NSCLCs is associated with the CSC content

**Figure 1:**
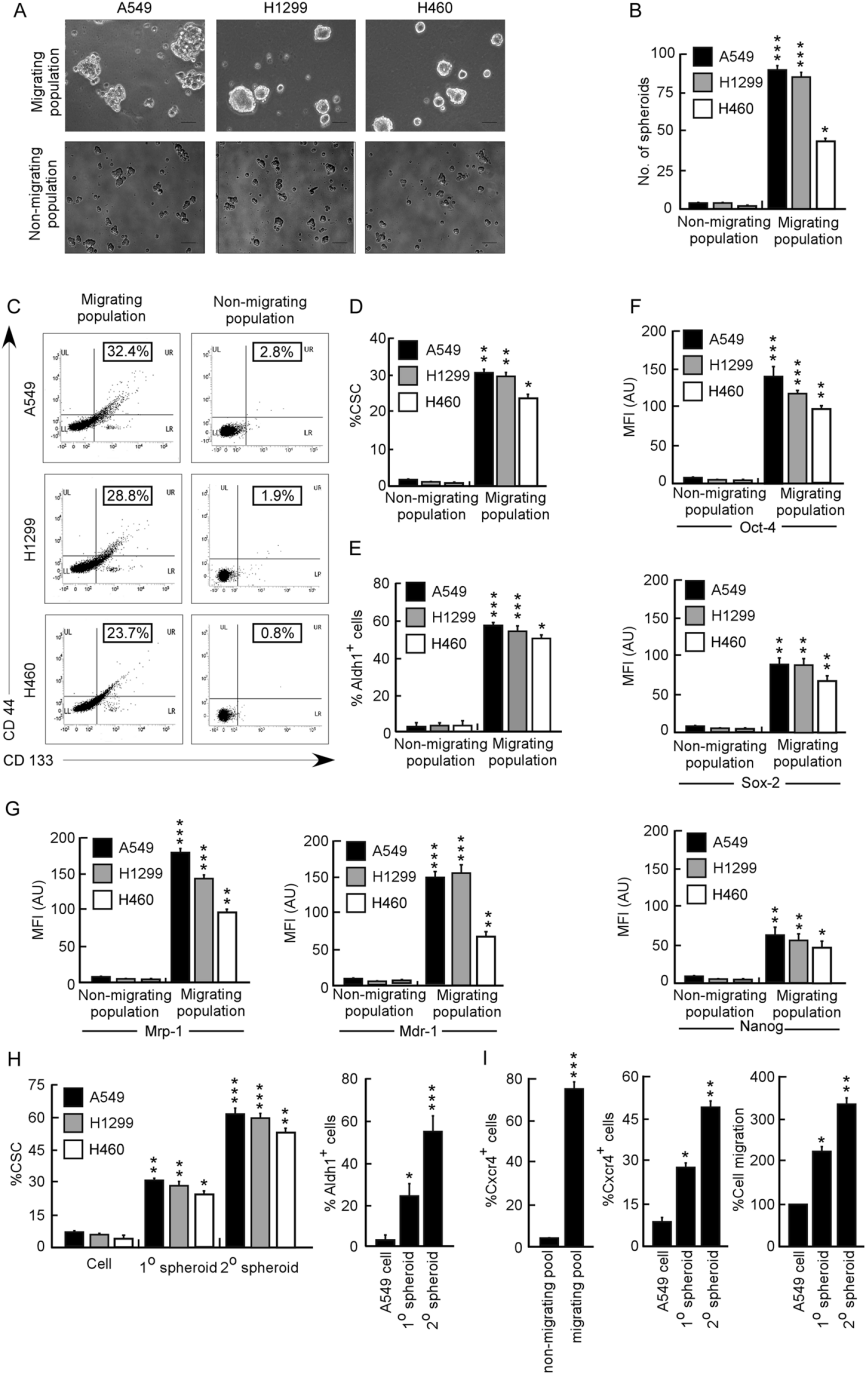
Migration potential of NSCLCs is linked to the CSC content. (**A**) Phase contrast images of spheroid formation assay from both migrating as well as non-migrating population of A549, H1299 and H460 cells. Scale bar 100 µm. (**B**) No. of spheroids were counted and graphically represented from both migrating as well as non-migrating population of A549, H1299 and H460 cells. (**C**) Representative plot of migrating as well as non-migrating population of A549, H1299 and H460 cells that were flow- cytometrically analysed for CD133/CD44-positivity and (**D**) was represented as bar plot. (**E**) Graphical representation of percent Aldh1-positive cells in both migrating as well as non-migrating population of A549, H1299 and H460 cells. (**F**) Relative mean fluorescence intensities of Oct-4, (upper panel) Sox-2 (middle panel) and Nanog (lower panel) and (**G**) Mrp-1 and Mdr-1 in both migrating as well as non- migrating population of A549, H1299 and H460 cells. (**H**) Graphical representation of flow-cytometric plots showing percent CSC (left panel) and percent Aldh1 positivity (right panel) in 2° spheroids, 1° spheroids and corresponding parental cells of A549, H1299 and H460. (**I**) Representative graphical illustration of percent CXCR4 positive cells in both migrating and non-migrating population of A549 cells (left panel). Graphical representation of percent CXCR4 positive cells in 2° spheroids, 1° spheroids and corresponding parental A549 cells (middle panel). Graphical illustration of percent cell migration of 2° spheroids, 1° spheroids and corresponding parental A549 cells (right panel), as detected by transwell migration assay. Values are mean ± SEM of three independent experiments in each case or representative of typical experiment. *p < 0.05, **p < 0.01, ***p < 0.001.

In view of the notion that CSC population is mostly responsible for tumor aggressiveness^21^, we used publicly available Oncomine database (www.oncomine.org) to obtain an overview of expressions of human lung cancer stem cell markers CD44 (Supplementary Fig. S1A,B) and CD133 (Supplementary Fig. S1C-E) in clinical specimens which demonstrated higher expression of both the markers in lung adenocarcinoma compared to control samples. Using the same database we also checked stage-wise involvement of CD133 in human lung adenocarcinoma and surprisingly the results indicated gradual increase of CD133 expression with disease progression of NSCLCs (Fig. S2A–E). Since recent reports^22–24^ have associated CSCs with metastasis and invasion; we next explored whether the migration potential is associated with the CSC content of the tumor. At this juncture, we performed spheroid formation assay from both migrating as well as non-migrating pool of A549, H1299 and H460 cells. Results showed that while the migrating population could form spheroids in serum-free stem cell culture media (Fig. 1A, upper panel, and Fig. 1B), the non-migrating population failed to do so (Fig. 1A, lower panel, and Fig. 1B). Moreover, the expressions of CD133, CD44 (Fig. 1C,D) and aldehyde dehydrogenase-1 (ALDH1) (Fig. 1E), were more in migrating fraction of A549, H1299 and H460 cells in comparison to the non-migrating one. In addition, there was increase in the expression levels of pleuripotency markers, Oct- 4, Sox-2, and Nanog (Fig. 1F), as well as drug-resistance markers, Mrp-1 and Mdr-1 (Fig. 1G) in these migrating populations of A549, H1299 and H460 cells than the corresponding non-migrating one. These results confirmed the presence of CSCs in the migrating population of NSCLC cells. Next, our effort to further validate the status of CSCs in spheroids of human NSCLC cells revealed that when cultured in serum-free stem cell culture media for 7 days, A549 cells formed typical spheroids (1° spheroid^25^, a marker of the self-renewal of stem cells^5^. When these 1° spheroids were re-cultured in serum-free stem cell culture media for 7 more days, 2° spheroids was formed and the expressions of CD133, CD44 (Fig. 1H left panel) and Aldh1 (Fig. 1H right panel) were increased in the order of parental cells < 1° spheroids < 2° spheroids. These results support the enrichment of CSCs in these 2° spheroids. In addition, in line with the reports that have positively correlated Cxcr4 expression with NSCLC cell migration^26,27^, we observed significantly higher expression of Cxcr4 in migrating population, containing higher number of CSCs (Fig. 1C,D), than the non-migrating one of A549 cells (Fig. 1I left panel). Next we evaluated the status of Cxcr4 in the CSC-enriched spheroids and in line with the report of Beck *et al*. and Tu *et al*.,^28,29^ higher expression of Cxcr4 in comparison to parental A549 cells as well as corresponding 1° spheroids, was also documented in these CSC-enriched A549 2° spheroids (Fig. 1I middle panel) that also furnished higher migration potential than the other two (Fig. 1I right panel). These results raised the possibility of a relationship between CSC content and NSCLC migration.

### CSCs exhibit enhanced Akt activation that leads to EMT

**Figure 2:**
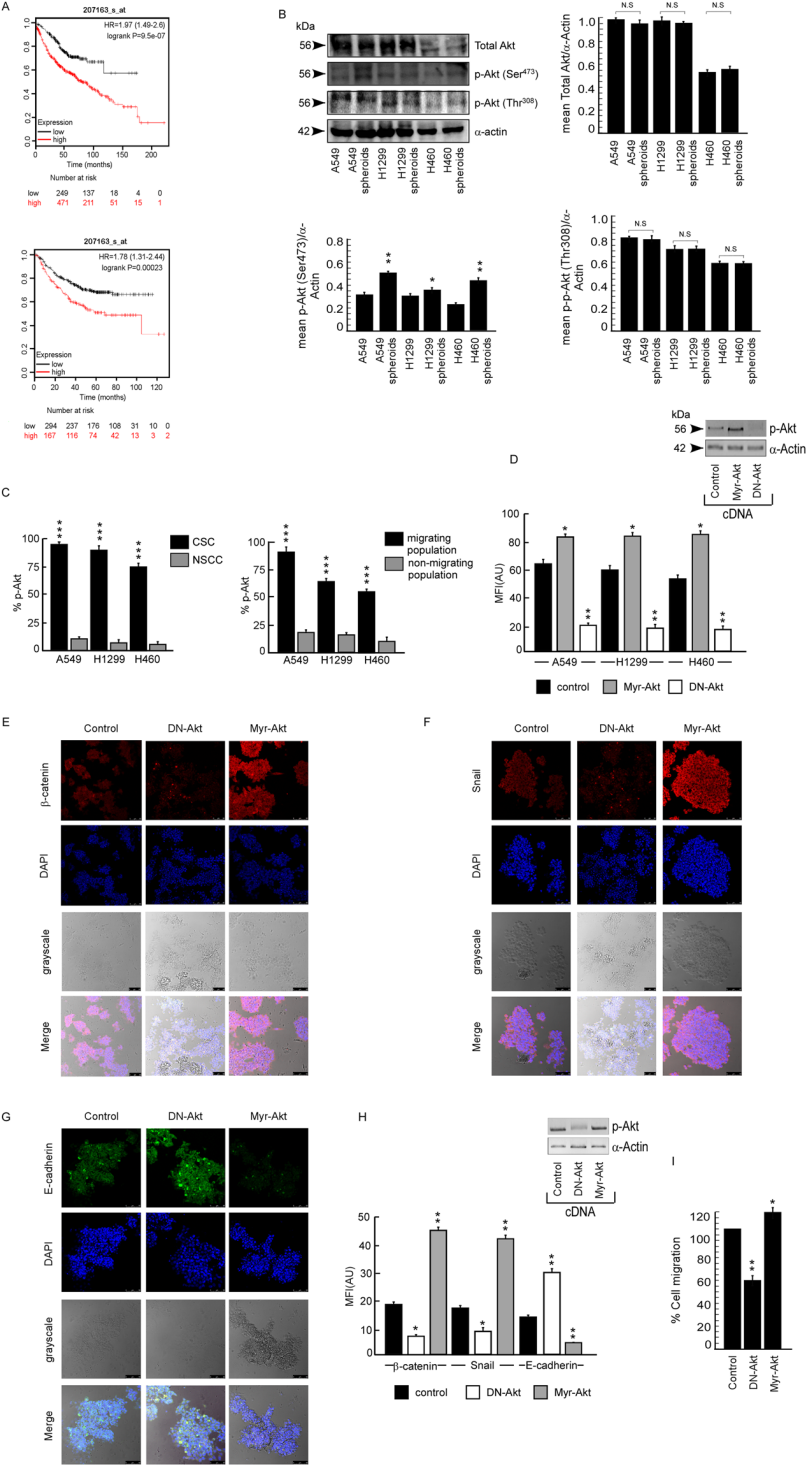
CSCs exhibit enhanced Akt activation that leads to EMT. (**A**) Kaplan-Meier Analysis Plots representing correlation of Akt expression with survival of first progression (FP) (upper panel) or overall survival (OS) (lower panel) of lung cancer patients. For analysing the prognostic value, the patient samples have been split into two groups according to various quantile expressions of Akt. Next, the two patient cohorts have been compared; the hazard ratios (HR) with 95% confidence intervals and logrank P value have been shown. (**B**) Cell lysates from A549, H1299 and H460 cells and corresponding spheroids were subjected to Western blot analysis for protein expression of total Akt, p-Akt (Ser^473^) and p-Akt (Thr^308^). α-Actin was used as loading control. Upper right, lower left and lower right panels show graphical representation of quantified band intensities. (**C**) Percent p-Akt expression was represented graphically as detected in flow-cytometrically gated CSC as well as NSCC population of A549, H1299 and H460 cells (left panel) and migrating as well as non-migrating population of the same (right panel). (**D**) Relative mean fluorescence intensities of CD133^+^/CD44^+^ population in control as well as DN-Akt and Myr-Akt transfected A549 spheroids. Transfection efficiency of DN- Akt and Myr-Akt were confirmed by immunoblots. α-actin was used as loading control. (**E**) Confocal microscopic images show the expression of β-catenin, (**F**) Snail and (**G**) E-cadherin in A549 control as well as DN-Akt and Myr-Akt transfected spheroids with DAPI staining for nucleus. Scale bar 50 µm; magnification 40×. (**H**) Relative mean fluorescence intensities of confocal images. (**I**) Graphical representation of percent cell migration as calculated in A549 control as well as DN-Akt and Myr-Akt transfected spheroids as detected by transwell migration assay. Values are mean ± SEM of three independent experiments in each case or representative of typical experiment *p < 0.05, **p < 0.01,***p < 0.001.

Since activation of Akt is considered to be responsible for tumor aggressiveness^30^, using publicly available database (KM plotter; mplot.com/analysis/index.php?p = service&cancer = lung), survival curves were analysed with reference to Akt expression (Fig. 2A) in lung adenocarcinoma, which indicated lower patient survival rate with higher Akt expression (analyses criteria explained in Material and Methods). In depth literature search also revealed hyper-activation of Akt as one of the major causes underneath greater migratory response of CSCs^31^. To validate the connection between activated Akt and migration of spheroids, we first checked the levels of phosphorylated Akt as well as total Akt in A549, H1299 and H460 cells and their corresponding spheroids. 2° spheroids were used for this and our further experiments with spheroids. Results of Fig. 2B demonstrate that spheroids derived from A549, H1299 and H460 cells possessed higher p-Ser^473^Akt expression, p-Thr^308^ and total Akt remaining unaltered, than their corresponding cell lines. In addition, flow-cytometrically gated CD133^+^/CD44^+^ populations from all the above-mentioned cell lines showed higher levels of Akt phosphorylation (p-Ser^473^) in comparison to corresponding CD133^−^/CD44^−^ non-stem counterpart (NSCCs) (Fig. 2C left panel). Role of Akt hyper-activation in migration of NSCLCs was further proved by our flow-cytometric analysis where migrating fraction of A549, H1299 and H460 cells, containing higher number of CSCs than non-migrating fraction (Fig. 1C,D), expressed higher levels of phospho- Akt (p-Ser^473^) than the non- migrating fraction (Fig. 2C right panel). In fact, Myr-Akt-transfected spheroids (constitutively active Akt condition) furnished significantly higher stemness as was evident from the expression levels of CSC markers, CD133^+^/CD44^+^, as compared to control cDNA transfectants (Fig. 2D). On the contrary, A549 spheroids, derived from dominant-negative Akt (DN-Akt) transfectants showed decreased expression of these CSC markers (CD133^+^/CD44^+^) in comparison to the control ones (Fig. 2D). Myr-Akt-transfected spheroids also furnished up-regulation of mesenchymal markers like Snail and β-catenin and down-regulation of epithelial marker like E-cadherin (Fig. 2E–G), while DN-Akt-transfected A549 spheroids exhibited down-regulation of Snail and β-catenin and up-regulation of E-cadherin (Fig. 2E–G). MFI values for each image were quantitaed and represented in Fig. 2H. In addition percent cell migration was also counted and found to be down- regulated upon DN-Akt-transfection and up-regulated during Myr-Akt-transfection (Fig. 2I). These results together signify the importance of activated Akt in migration of cancer stem cells.

### Aspirin sensitizes spheroids towards the anti-migration effect of cisplatin

**Figure 3:**
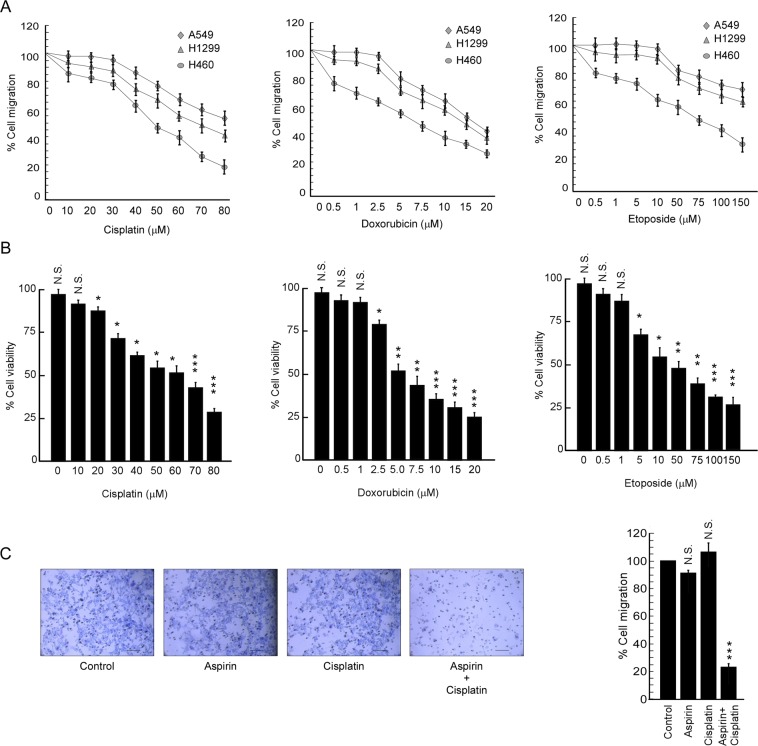
Aspirin sensitizes spheroids towards the anti-migration effect of cisplatin. (**A**) Graphical representation of the relative quantification of percent cell migration of A549, H1299 and H460 derived spheroids treated with different doses of cisplatin, doxorubicin and etoposide as detected by transwell migration assay. (**B**) Percent viability of PBMC treated with a dose range of genotoxic drugs, cisplatin, doxorubicin and etoposide, was scored by using trypan blue dye exclusion assay. (**C**) Representative bright field images of percent cell migration of A549 spheroids treated with 2.5 mM aspirin, 10 µM cisplatin, individually or in combination (left panel) and graphical representation of same (right panel). Scale bar 100 µm. Values are mean ± SEM of three independent experiments in each case or representative of typical experiment *p < 0.05, **p < 0.01,***p < 0.001.

Next we screened different conventional chemotherapeutic drugs like cisplatin, doxorubicin and etoposide, for their anti-migratory effects on CSC-enriched spheroids. Our result revealed that the sub-lethal doses of each of the drugs were unable to retard migration of A549 spheroids (Fig. 3A). Lethality was checked in peripheral blood mononuclear cells (PBMCs) (Fig. 3B). At this stage, since spheroids showed hyperactivation of Akt (Fig. 2A,B) and some recent studies reported that non-steroidal anti-inflammatory drug, aspirin, down-regulates mTOR, the kinase molecule upstream of Akt^32,33^, we explored the effect of aspirin on migration of A549 spheroids first. Surprisingly, aspirin alone failed to inhibit migration of spheroids as observed by transwell migration assay (Fig. 3C). This indulged us to consider whether aspirin in combination with lung cancer-specific conventional chemotherapeutic drug cisplatin can inhibit the same. Figure 3C revealed that pre-treatment of aspirin followed by exposure to non-toxic dose of cisplatin decreased the percent migration of A549 spheroids while each of these drugs alone failed to affect the same. At this juncture, we adopted a hypothesis that, although alone failing to block migration of CSCs, aspirin might sensitize the same towards anti-migratory effect of cisplatin.

### Aspirin inhibits mTOR/Akt axis to sensitize spheroids

**Figure 4:**
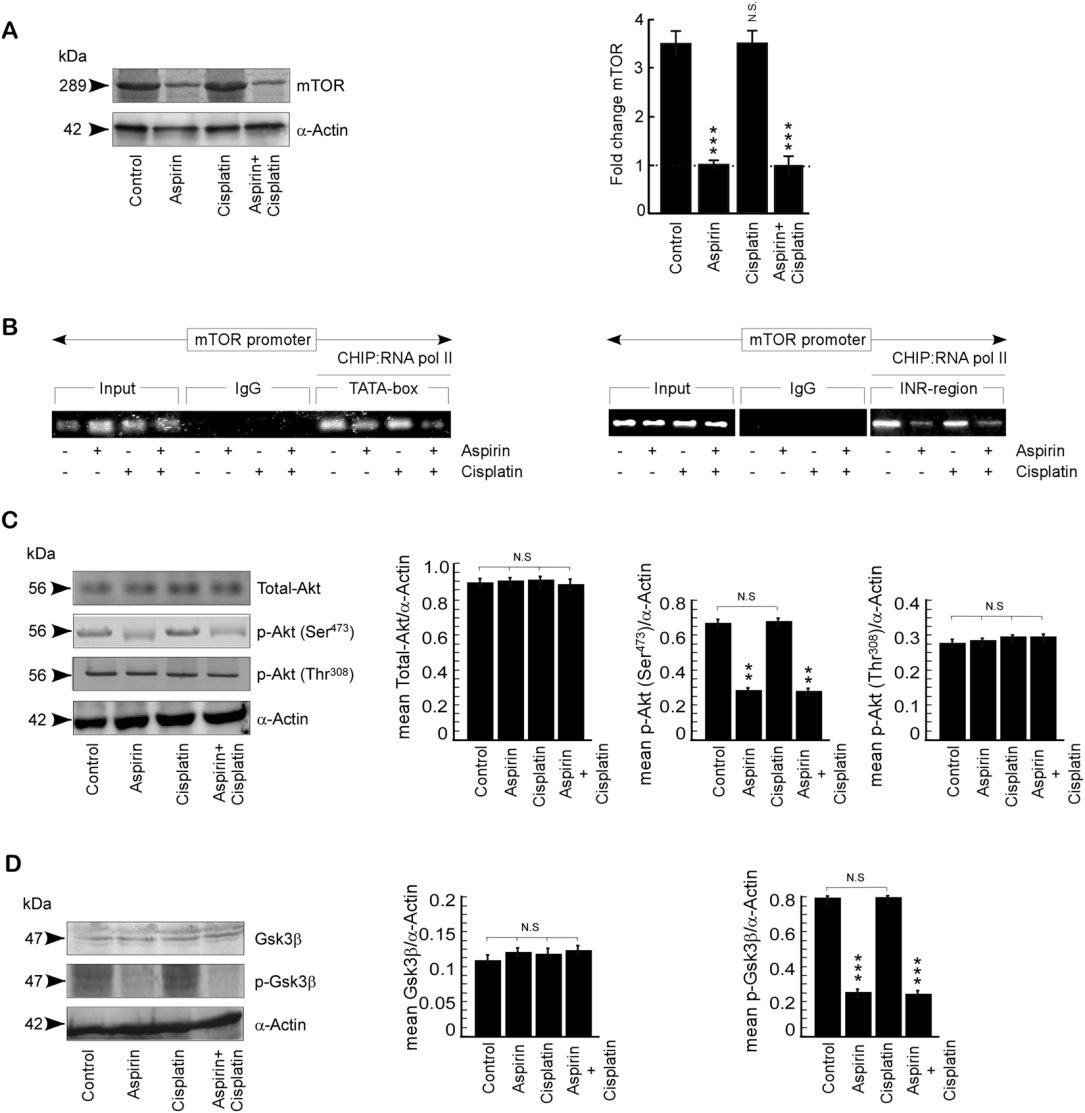
Aspirin inhibits mTOR/Akt axis to sensitize spheroids. (**A**) Protein expression profiles of mTOR (left panel) and graphical representation of the representative fold change value of mRNA level of mTOR (right panel) in un/-aspirin/-cisplatin/-aspirin + cisplatin treated A549 spheroids, as determined by Western blot and RT-PCR analyses respectively. α-Actin/ GAPDH served as loading controls. (**B**) ChIP assay for RNA pol II binding on TATA-box (left panel) and INR-region (right panel) of mTOR promoter in un/- aspirin/-cisplatin/-aspirin + cisplatin treated A549 spheroids. Chromatin from all these sets was immunoprecipitated with RNA pol II antibody followed by PCR amplification. (**C**) Western blot depiction of total-Akt, p-Akt (Ser^473^), p-Akt (Thr^308^) and (**D**) GSK3β and p-GSK3β in un/-aspirin/-cisplatin/-aspirin + cisplatin treated A549 spheroids. α-actin was used as loading control. Middle panels and right panel shows graphical representation of quantified band intensities. Values are mean ± SEM  of three independent experiments in each case or representative of typical experiment *p < 0.05, **p < 0.01,***p < 0.001.

To investigate further details of how aspirin enhances cisplatin sensitivity, we first checked whether aspirin inhibits mTOR/Akt axis in spheroids since these cells have been shown (Fig. 2C) high expression of p-Akt. Results of Fig. 4A demonstrate decrease in mTOR at both protein (Fig. 4A, left panel) and mRNA (Fig. 4A, right panel) levels by aspirin alone (second lanes) in comparison to control. However, cisplatin alone failed to show any effect on mTOR (Fig. 4A, left and right panels, third lanes). Interestingly, in the aspirin + cisplatin combination set (Fig. 4A, forth lane), decrease in mTOR levels similar to aspirin alone set was observed, which might solely because of the down regulation of mTOR by aspirin. Chromatin immunoprecipitation (ChIP) assay further showed that aspirin hinders the binding of RNA pol II on TATA-box region in addition to INR region of mTOR promoter (Fig. 4B). A search for the down-stream mechanism revealed significant reduction in Akt phosphorylation at Ser^473^while total Akt and phosphorylation at Thr^308^ of Akt remained unaltered (Fig. 4C). Furthermore, aspirin treatment decreased phosphorylation of Gsk3β, the downstream effector of Akt-p-Ser^473^, leaving total Gsk3β level unaltered (Fig. 4D). These results together indicated that aspirin pre- treatment sensitizes CSCs by jeopardizing mTOR/Akt axis.

### Aspirin impedes association of p65NFkB with p300 to down-regulate mTOR transcription

**Figure 5:**
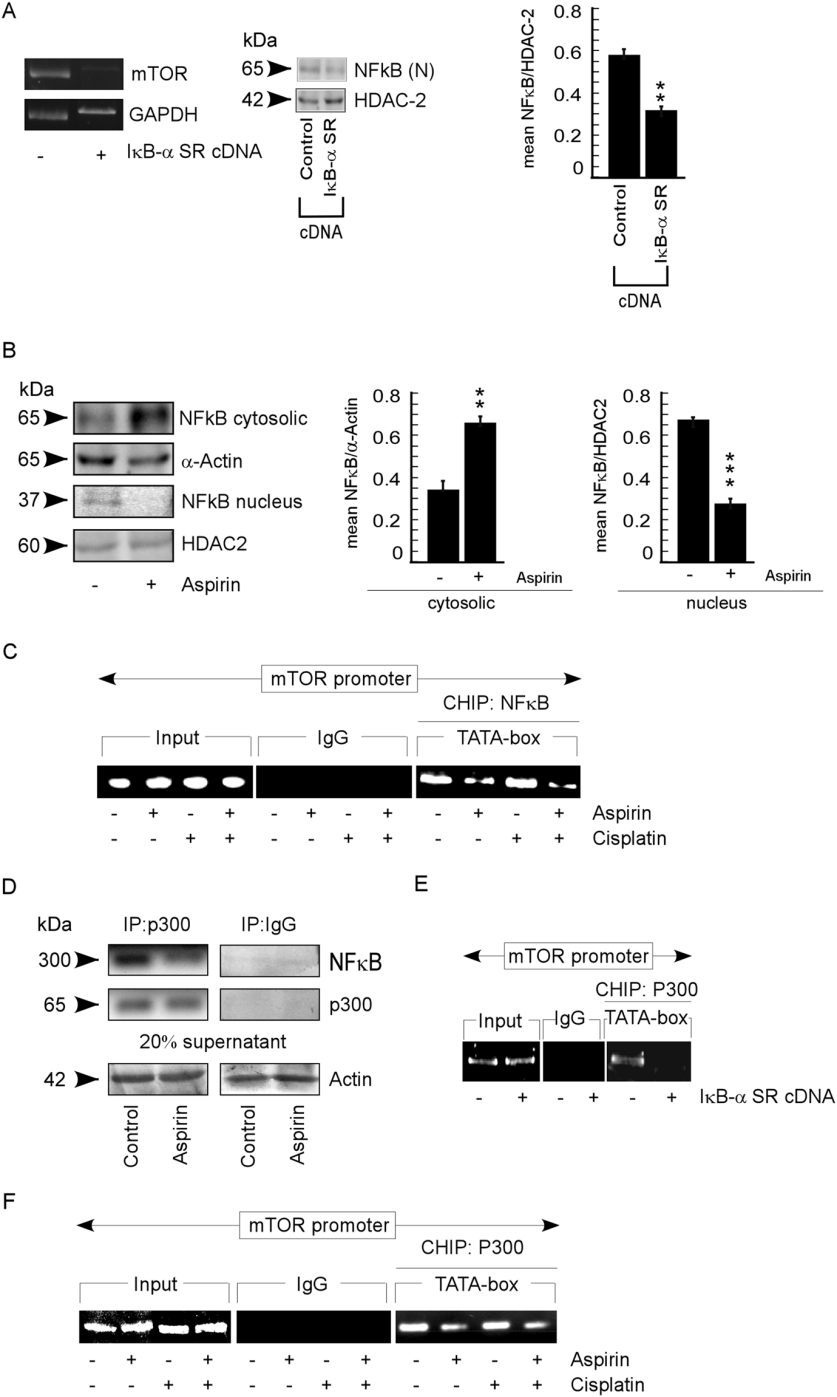
Aspirin impedes association of p65NFkB with p300 to down-regulate mTOR transcription. (**A**) RT-PCR analysis for mTOR mRNA levels in untransfected, IκBα-SR-cDNA- transfected A549 spheroids. The inset shows the immunoblot analysis of nuclear p65NFκB levels for the transfection efficiency of IκBα-SR-cDNA. HDAC2 served as loading control for nuclear lysate. Right panel shows graphical representation of quantified band intensities. (**B**) Protein expression levels of p65NFκB in nuclear and cytosolic lysates of untreated and aspirin treated-A549 spheroids as determined by western blot assay. α-Actin served as loading control in cytosolic lysate and HDAC2 served as loading control for nuclear lysate. Middle panel and right panel show graphical representation of quantified band intensities of cytosolic and nuclear lysates respectively. (**C**) ChIP assay for p65NFκB binding on the TATA-box region of mTOR promoter in untreated or aspirin or/and cisplatin treated A549 spheroids. (**D**) Nuclear lysates of untreated or aspirin treated A549 spheroids were subjected to immunoprecipitation using an anti- p300 antibody or with control IgG. The same blot was sequentially probed with p65NFκB andp300-specific antibodies. To confirm comparable protein input of the respective samples following, 20% of lysates used for immunoprecipitation were subjected to western blot analysis with anti-α-Actin. (**E**) ChIP assay for p300 binding on the TATA-box region of mTOR promoter in untransfected, IκBα-SR- cDNA transfected A549 spheroids and (**F**) ChIP assay for p300 binding on the TATA-box region of mTOR promoter in untreated or aspirin or/and cisplatin treated A549 spheroids. Values are mean ± SEM of three independent experiments in each case or representative of typical experiment *p < 0.05, **p < 0.01, ***p < 0.001.

Since aspirin inhibits the nuclear localization of active NFκB^13^, we further verified whether this effect of aspirin was instrumental in reducing the binding of RNA pol II to *mTOR* promoter. To that end, first we examined whether p65NFκB binding was essential for mTOR transcription to occur. Our results showed that when p65NFκB nuclear translocation was inhibited by transfecting spheroids with IκBα super-repressor cDNA (IκBα-SR-cDNA), mTOR transcription was down-regulated (Fig. 5A). Similarly aspirin treatment restrained p65NFκB nuclear translocation in these cells (Fig. 5B), along with the decrease in p65NFκB binding on TATA-box region of mTOR, as confirmed by ChIP analysis (Fig. 5C). These results indicate that aspirin hinders mTOR transcription by inhibiting the translocation of p65NFκB to the nucleus, which is a pre-requisite for mTOR expression to occur.

Since according to Sen *et al*.^34^, p65NFκB recruits histone acetyletransferase 1 (HAT1), i.e., p300, to DNA, we checked the acetylation status of *mTOR* promoter to understand the exact molecular mechanism underlying chromatin modification in our system. At this juncture we hypothesized that p65NFκB is recruiting p300 for acetylating *mTOR* promoter to start and proceed for mTOR transcription successfully. Confirming our hypothesis, our co-immunoprecipitation experiment demonstrated association between p65NFκB and p300 in untreated spheroids (Fig. 5D) and such association was decreased in aspirin-treated set (Fig. 5D). To confirm the binding efficacy of p300 on the mTOR promoter in the presence or absence of p65NFκB, IκBα-SR-cDNA was transfected in spheroids. The results showed decreased acetylation on the TATA-box region of mTOR promoter in IκBα-SR-cDNA transfectants (Fig. 5E). Finally, aspirin decreased the acetylation of *mTOR* promoter which remained same when treated with cisplatin alone (Fig. 5F), thereby validating our hypothesis that aspirin decreases mTOR transcription by inhibiting nuclear translocation of p65NFκB, which otherwise recruits p300 for chromatin acetylation and promotes subsequent binding of RNA pol II on *mTOR* promoter to proceed transcription effectively.

### Downregulation of mTOR-Akt axis by aspirin inhibits EMT-promoting factors in spheroids

**Figure 6:**
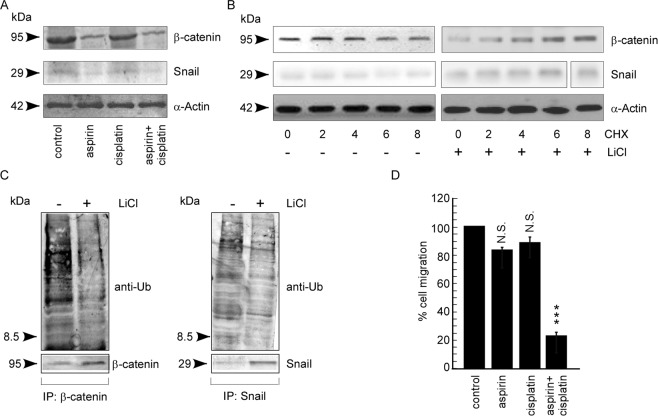
Downregulation of mTOR-Akt axis by aspirin inhibits EMT-promoting factors in spheroids. (**A**) Western blot analysis to determine β-catenin and Snail expression levels in untreated or aspirin/-cisplatin/-aspirin + cisplatin treated A549 spheroids. (**B**) Protein expression levels of β-catenin and Snail of untreated and CHX and/or LiCl-treated lysates in spheroids as determined by western blot assay. α-Actin served as loading controls. (**C**) β-catenin (left panel) Snail (right panel) immunoprecipitates from un/-LiCl-treated A549 spheroids were probed with anti-ubiquitin antibodies to determine the levels of β-catenin, or Snail degradation respectively (upper panels). Total β-catenin, or total Snail level was determined in un/-LiCl-treated spheroids (lower panels). (**D**) Graphical representation of percent cell migration in untreated or aspirin or/and cisplatin treated A549 spheroids as observed by transwell migration assay. Values are mean ± SEM of three independent experiments in each case or representative of typical experiment *p < 0.05, **p < 0.01,***p < 0.001.

Considering that (i) activated Gsk3β targets EMT-promoting molecules, e.g., β-catenin^10^, and Snail^11^ (ii) aspirin treatment dephosphorylates Gsk3β (Fig. 4D) thereby activating it in spheroids, we next assessed the status of the above-mentioned target molecules and their link to migration in our experimental system. The protein levels of β-catenin and Snail were decreased upon treatment of aspirin alone (Fig. 6A, second lane), while cisplatin alone furnished no such effect on these protein expression levels (Fig. 6A, third lane) in comparison to control. In the combination set (Fig. 6A, forth lane), decreases observed in β catenin and snail levels were similar to those observed with aspirin alone and thus might be due to their down-regulation by aspirin only.

To re-confirm the effect of Gsk3β phosphorylation on β-catenin and Snail in spheroids, these cells were treated with lithium chloride (LiCl), a well-known inhibitor of Gsk3β^35^, to mimic the condition of Akt-induced inhibition of Gsk3β (Fig. 6B). The results of Fig. 6B depicted LiCl-induced stabilization of these EMT- promoting proteins in A549 spheroids, thereby supporting our conception that while inactivation of Gsk3β upon Akt activation stabilized EMT-promoting factors β-catenin and Snail, upon aspirin-mediated de-phosphorylation and activation of Gsk3β, these proteins might have undergone degradation, thereby showing decreasing trends in expression (Fig. 6A). At this juncture, A549 spheroids were treated with cyclohexamide (CHX), the inhibitor of protein biosynthesis, with or without LiCl (Fig. 6B). The results of Fig. 6B furnished that while treatment of CHX alone decreased β-catenin and Snail, combination of CHX treatment and LiCl stabilized them. These results led us to the conclusion that decrease in the levels of these EMT-promoting proteins in the absence of new protein synthesis might be due to Gsk3β-induced degradation, which Gsk3β inhibitor LiCl resisted the same. Additional support came from the experiment in which in comparison to control spheroids, decrease in co-immuno-precipitation of β-catenin/Snail with ubiquitin in LiCl-treated set indicated stabilization of these three proteins upon Gsk3β inactivation (Fig. 6C). These results, therefore, extend the possibility of aspirin- mediated inactivation of EMT program in CSCs by degradation of these EMT-promoting factors.

Although aspirin treatment altered the expression of EMT-promoting factors in spheroids, it failed to furnish evidence for any significant decrease in migration potential of the same (Fig. 3C). However, pre-treatment of aspirin followed by cisplatin hindered the same (Fig. 3C). It can, therefore, be hypothesized that aspirin pre-treatment, although could not bring out the final anti- migration effect, it might have altered the intra-cellular micro-environment by re-arranging the above- mentioned molecular architecture of spheroids in favour of cisplatin’s anti-migration effect. Therefore, next we aimed at validating our hypothesis by deciphering the signalling pathway by which cisplatin ultimately imposes its anti-migratory effect in aspirin-sensitized CSCs.

### Cisplatin impedes migration by deterring Integrin/Fak signaling in aspirin pre-treated spheroids

**Figure 7:**
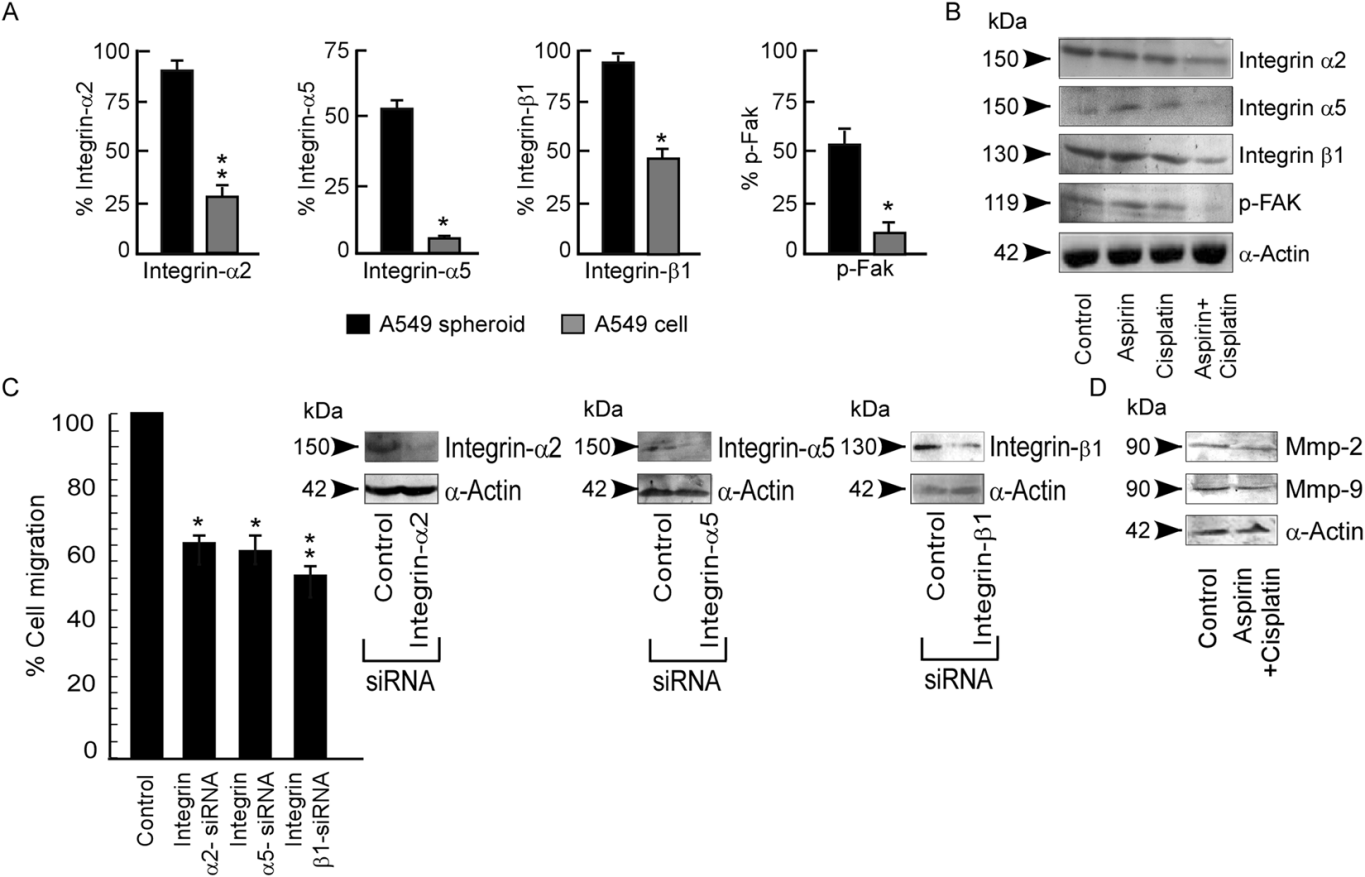
Cisplatin impedes migration by deterring Integrin/Fak signalling in aspirin pre-treated spheroids. (**A**) Percent Integrin-α2 (left panel), Integrin-α5 (middle panel) and Integrin-β1 (right panel) expression was represented graphically as detected by flow-cytometry in A549 parental cell and spheroids. (**B**) Western blot analysis of Integrin-α2, Integrin-α5, Integrin-β1 and p-FAK in untreated or aspirin/-cisplatin/-aspirin + cisplatin treated A549 spheroids. α-Actin was used as loading control. (**C**) Percent cell migration was calculated and represented graphically in control and Integrin-α2-siRNA, Integrin-α5–siRNA or Integrin-β1-siRNA transfected A549 spheroids as detected by transwell migration assay. The inset shows the immunoblot analysis of Integrinα2, Integrinα5 or Integrinβ1 levels for the transfection efficiency of Integrin-α2-siRNA, Integrin-α5–siRNA and Integrin-β1-siRNA respectively. (**D**) Protein expression levels of MMP-2 and MMP-9 in untreated or aspirin + cisplatin treated A549 spheroid. α-Actin served as loading control. Values are mean ± SEM of three independent experiments in each case or representative of typical experiment *p < 0.05, **p < 0.01, ***p < 0.001.

Considering (i) our previous results that CSCs are resistant to the anti-migratory effect of cisplatin (Fig. 3A), and (ii) the report that cisplatin-resistant cell lines over-express Integrin/Fak^15–17^, we next explored the status of Integrin/Fak in A549 spheroids and the effect of aspirin on these proteins. Untreated A549 spheroids showed higher levels of Integrin-α2, Integrin-α5, Integrin-β1 and p-Fak in comparison to corresponding parental cell (Fig. 7A), and while aspirin treatment failed to suppress Integrin-α2, Integrin-α5, Integrin-β1 and p-Fak expression in spheroids, cisplatin treatment in combination could restrain the same (Fig. 7B). These results, therefore explain the cause underlying the failure of aspirin in hindering migration of A549 spheroids. Further results showed that the percent migration decreased sharply in Integrin-α2/Integrin-α5/Integrin-β1 siRNA- transfected A549 spheroids (Fig. 7C) indicating the involvement of Integrin signaling in migration of these cells. In line with the report that mTOR inhibition restores cisplatin sensitivity^36^, we also observed that, in aspirin pre-treated A549 spheroids, along with Integrin-α2, Integrin-α5, Integrin-β1 and p-Fak (Fig. 7B), cisplatin also decreased Mmp-2 and Mmp-9 expression (Fig. 7C), thereby halting migration of combinatorial therapy-treated CSCs. As a result, EMT was reversed and migration was inhibited in combinatorial therapy-treated CSCs.

The entire work together explore the previously unrevealed role of FDA-approved drug aspirin in perturbing metastatic potential of CSCs, by sensitizing them towards the anti-migration effect of commonly used chemotherapeutic drug cisplatin. These findings raised the possibility of repurposing of aspirin in combinatorial therapy for reducing the probability of metastasis in drug-resistant NSCLC patients.

All these results accumulatively indicate that aspirin in combination with cisplatin is capable of abrogating metastasis in non-small cell lung carcinoma stem-like cells.

## Discussion

Metastasis to other secondary organs in NSCLC patients is the most intricate challenge for the efficacious treatment and thus successful eradication of the disease. The series of experimentations sketched in this report focuses on cisplatin since it is one of the major drugs in the standard chemotherapy schedule prescribed for treatment of NSCLCs. In reality, almost 63% NSCLC patients are resistance to cisplatin treatment and over 68% of patients show resistance with carboplatin treatment^37^. Mechanisms that majorly influence resistance include generation of metastatic tumor^22,23^, reduced uptake and/or augmented efflux^4^, improved DNA repair^38^, and collapse of cell-death pathways^38^. Chemoresistance and metastasis are reported to be closely correlated in human cancer patients^39^. In metastatic setting marked decrease of chemotherapy response rate indicate that in comparison to primary tumors, metastatic tumors are consistently more resistant to chemotherapy^39^. Furthermore, recent literatures have shown a clear connection between chemotherapy resistance and the EMT phenotype^39^. In this regard, it is observed that NSCLC cell lines undergoing EMT are resistant to cisplatin^39^. Like other researchers, we also found that non-toxic doses of conventional chemotherapeutic drugs like cisplatin, doxorubicin and etoposide, showed incapability to retard migration of CSCs derived from different NSCLC cell lines. Therefore, treatment of resistant cancers particularly needs therapeutic combinations using EMT-signaling inhibitors. Therapy-resistant CSCs might be accountable for such failure of platin-based conventional therapies. Hence, distinct CSC inhibitors may, appear as the promising anti-tumor agents for successful abolition of cancer. In this study, we have unveiled the inimitable role of aspirin, an FDA approved NSAID, in sensitizing CSCs. Lately, there has been renewed attention in repurposing aspirin and its derivatives as anticancer agents because of their capability to distinctively inhibit Cox-1/Cox-2 and NFκB pathway, and to down-regulate numerous genes mediating proliferation, chemo-resistance, invasion/metastasis and angiogenesis of cancer cells. Yet, information detailing the effect of aspirin in migration of CSCs are lacking presently.

According to Fraser *et al*.^40^, cisplatin-induced chemoresistance occurs by retardation of p53, which ultimately direct to down-regulation of PTEN and activation of Akt in human ovarian carcinoma. Previously, Jiang *et al*.^41^ found augmentation of Akt1 increases laryngeal squamous cell carcinoma (LSCC) cell growth and migration. Furthermore, CSCs are also reported to have Akt hyper-activation and thus resulting in potentiation of the PI3K/mTOR/Akt signaling pathway^31^. In our study, we also observed that CSCs derived from NSCLCs also exhibit Akt hyper-activation and mTOR over- expression as well. In line with this, Sunayama *et al*.^8^ established that cross-inhibitory modulation between Mek/Erk and PI3K/mTOR pathways regulates the maintenance of the self-renewal as well as the tumorigenic capacity of glioblastoma cancer stem-like cells. Accordingly, Corominas-Faja *et al*.^42^ used modified stem cell technology of Yamanaka *et al*.^42^ in an attempt to construct stable CSC lines, and they observed the transcriptional suppression of mTOR repressors to be a fundamental procedure occurring in luminal-like breast cancer cells throughout the acquisition of CSC-like properties. These results indicate the possibility that stimulation of mTOR signaling could induce the generation of CSCs. Although in sharp contrast, a controversial report by Yang *et al*.^43^ says that, mTOR inhibition by rapamycin not only increased the CD133^+^ subpopulations in totality, but also triggered the conversion of CD133^−^ to CD133^+^ significantly, in gastrointestinal cancer cells. Yet there are several reports which state that suppression of mTOR decreases activity of Aldh1 as well as EpCAM, the well-known markers for cancer stem cells^44^. In line with this, Mateo *et al*.^45^, showed that, in breast cancer stem-like cells activation of the mTOR pathway is necessary for*in vitro* colony-formation ability and *in vivo* tumorigenicity. Aspirin, however, have shown its potential to down-regulate mTOR expression in our study. In fact, aspirin halts mTOR transcription by inhibiting binding of RNA polymerase II at mTOR TATA-box region as well as INR region. Downregulation of mTOR, which is the upstream kinase molecule of Akt, in turn leads to dephosphorylation of Akt at Ser^473^ and its deactivation, thereby resulting in Gsk3β activation. This was evident by Case *et al*.^46^, who showed that in mTORC2 blocked condition, mechanical Gsk3β inactivation was prevented and the mechanical regulation of Gsk3β was reliant on phosphorylation of Akt at Ser^473^. In line with this, Zeng *et al*.^47^ demonstrated that, phosphorylated Gsk3β gets inactivated by activated Akt, and thus stabilizes the unphosphorylated β-catenin, which after getting translocated into the nucleus activates the target genes that regulate tumor metastasis and support cell survival. They also described about a novel signaling pathway that controls the stability of snail protein through Gsk3β-mediated phosphorylation^11^. In line with these, canonical Wnt signaling pathways are reported to prevent GSK3β-dependent phosphorylation and subsequent ubiquitination followed by proteasomal degradation of snail protein. As a result, snail protein level gets increased and activates snail- dependent EMT programs^47^. In our study we have observed that, activation of GSK3β upon aspirin treatment, leads to degradation of snail and β- catenin.

To our surprise, aspirin treatment could not retard migration of CSCs, inspite of inducing degradation of mesenchymal markers - β-catenin and snail. This fact indulged us to study the expression and activation of other proteins like integrins and Fak which are also responsible for EMT. Expression of Integrin-α2, Integrin-α5, Integrin-β2 and activation of Fak was observed to remain unchanged upon aspirin treatment. Therefore, it can be assumed that aspirin, though alters expression of β-catenin and snail, could not abrogate metastasis of CSCs; since they are unable to alter the status of these proteins. We, therefore, aimed at verifying whether combinatorial treatment of aspirin with cisplatin can abrogate metastasis of CSCs at a non-toxic dose. There are some reports saying that cisplatin-resistant cell line shows activation of Intrgrin-Fak signaling pathway^15^. Likewise, we observed that cisplatin treatment alone could not down-regulate the expression of Integrins and deactivate Fak in turn. However, combinatorial treatment of aspirin and cisplatin altered the same as well. It is, therefore, indicated that aspirin sensitizes the cellular microenvironment towards the chemotherapeutic drug cisplatin to retard migration of CSCs. Our results are in line with those by Wang *et al*.^48^ who reported that aspirin decreased ALDH1^+^ cell number in the total colorectal cancer cell population thus decreasing colonosphere formation. Since in keeping with the cancer stem cell theory, ALDH1^+^ CSCs are responsible for tumor initiation and migration^49^, results of Wang *et al*. support our hypothesis that aspirin changes the pro-migratory intracellular microenvironment to the anti-migratory one. Further validation of our findings came from the report of Zhang *et al*.^50^, who demonstrated that aspirin reduced invasion of pancreatic tumors in orthotopic mice xenografts in combination with gemcitabine. Another study by Lucotti *et al*.^51^ also documented that aspirin inhibits the formation of metastatic intravascular niches thus decreasing the number of metastatic lung nodules in experimental mice model and signifying an extensive inhibitory effect of this drug on metastasis.

Taken as a whole, the findings of this study clearly reveal the efficiency of aspirin to sensitize CSCs to overcome drug-resistance potential of them as well as to accelerate the capability of chemotherapeutic drug cisplatin to retard metastasis. This combinatorial strategy, therefore, may open a new window for more effectual cure for treatment-resistant lung carcinomas.

## Experimental Procedures

### Cell culture and treatments

We have procured non-small cell lung cancer cell line A549 (K-ras v-12 mutated) and H460 from National Centre for Cell Science, Pune, India. Dr. Susanta Roychoudhury (Saroj Gupta Cancer Centre and Research Institute, India) kindly gifted H1299 in 2015. Cell lines were regularly authenticated by short-tandem repeat analysis and passaged for less than 6 months after resuscitation^52^. The cells were routinely maintained in complete Dulbecco’s Modified Eagle’s Medium at 37 °C in a humidified incubator containing 5% CO_2_^52^. Before experiments, the cells were allowed to reach confluency. All migration assays were carried out in the presence of 10 μg/mL mitomycin C to negate the effect of cell proliferation^53^.

### Spheroid culture

For spheroid culture, A549 cells were seeded at 2.5 × 10^4^ cells per well in six well Ultralow Adherence plates (Corning Inc., Corning, NY, USA) in DMEM/F12 with 5 μg/mL bovine insulin (Sigma-Aldrich), 20 ng/mL recombinant epidermal growth factor, 20 ng/mL basic fibroblast growth factor, 0.4% bovine serum albumin (BSA) and B27 supplement (BD Biosciences, San Jose, CA, USA), and as previously described^54^. Primary/1° and secondary/2° spheroid formation was achieved by using weekly trypsinization and dissociation followed by reseeding in spheroids media at 2.5 × 10^4^ cells per well into Ultralow Adherence sixwell plates^54^.

### Treatment of cells

Cells were subjected to treatment with different concentrations of aspirin (MP Biomedicals) and cisplatin (Calbiochem) for different time points in order to optimize the dose and time necessary to reduce cancer stem cell migration. Untreated cells were subjected to analogous amount of carrier (dimethyl sulfoxide)^4^.

### Transwell migration assay

Transwell migration assay was carried out using 8.0-μm cell culture inserts (BD Biosciences) for assessing migration potential of A549, H1299 and H460 cells, and their corresponding 1° and 2° spheroids. For the same, cells were plated at 2.5 × 10^5^ cells (either parental or 1° or 2° spheroids) per well in serum-free DMEM in the upper chamber of 12-well plates and allowed to migrate for 24 hours toward DMEM containing 10% FBS in the lower chamber. After 24 hours, to determine percent cell migration the cells in the upper chamber were collected by removing with a cotton swab and the cells that have migrated in the lower surface of the membrane were fixed and stained with giemsa. A brightfield microscope (Leica, Wetzlar, Germany) was used acquire images at 20x magnifications. Migratory cells were quantified by analyzing three independent fields using ImageJ software (National Institutes of Health, Bethesda, MD, USA)^22^. Migration was expressed as percentage of cells migrated. For the same, the percentage of cells that migrated in the control set of each relevant experiment was taken as 100%^22^. For co-culture experiment and to pool migrated and non-migrated fraction of 2° spheroids, both fraction were collected from the upper as well as the under-surface of the membranes after 24 hour of migration either for reseeding meant for new experiment or for flow cytometry^22^.

### Flow cytometry

Expression of human NSCLC stem cell marker CD133 and CD44 was analyzed by flow cytometric study in A549 cells and spheroids by using anti-CD133-FITC and anti-CD44-APC antibodies (BD Biosciences). CD133^+^ and CD44^+^ CSCs were flow-cytometrically gated from spheroids on the basis of the cell surface phenotype. Mean fluorescence intensities of Oct-4-PerCP-Cy5.5, Nanog-PE, Sox-2- Alexa Fluor-647, Mrp1-FITC, Aldh1-FITC (BD Biosciences) were quantified^22^. Mean fluorescence intensities of Akt, p-Akt, Cxcr-4, Mdr1, Integrin-α2, Integrin-α5, Integrin-β1, p-Fak (Santa Cruz Biotechnology, Inc.) were determined with respective primary antibodies conjugated with PE as previously described^13^.

### Immunofluorescence

The living spheroids were fixed in 4% paraformaldehyde, permeabilized in 0.1% Triton X-100, and blocked in blocking buffer (10% BSA in PBS)^55^. Following the method used by Saha *et al*.^54^, spheroids were then incubated with 1.5% BSA containing anti- CD133/CD44/p-Fak/Integrin-α5/Integrin-α2/Integrin-β1 antibody (Santa Cruz, CA, USA). After washing in PBS, cells were incubated with secondary antibodies conjugated with FITC (green) or PE (red). The DAPI was used as nuclear stain (blue). Images were acquired using a confocal microscope (Carl Zeiss, Jena, Germany)^54^.

### Immunoblotting

Whole cell lysates were obtained by homogenizing, A549 cells and CSC-enriched spheroids in lysis buffer (20 mM Hepes, pH 7.5, 1.5 mM MgCl_2_, 10 mM KCl 1 mM Na-EDTA, 1 mM DTT and 1 mM Na-EGTA) supplemented with phosphatase and protease inhibitor cocktails. Western blot analysis was performed by resolving a total of 50 μg of protein with the help of SDS-PAGE and transferring to nitrocellulose membrane. This was further probed with specific antibodies^31^, for example, anti- Akt/- p-Akt (Ser473)/- p-Akt (Thr^308^)/- mTOR/- GSK-3β/- p- GSK-3β/-NFκB/- p300/- β-catenin/- snail/- slug/- Ub/- Integrin- α2/- Integrin- α5/- Integrin-β1/- p-FAK/- Integrin-α2/- Mmp-2/- Mmp-9 antibodies (Santa Cruz, CA, USA), thereafter the immunoblots were visualized by chemiluminescence (GE Biosciences, NJ, USA)^13^. α- actin or HDAC-2 was used as loading control^13^.

### RT–PCR assay

Two µg of the total RNA, extracted from spheroids with TRIzol Q16 reagent (Invitrogen, Carlsbad) was reverse transcribed and then subjected to PCR with enzymes and reagents of the RTplusPCR system (Eppendorf, Hamburg, Germany) using GeneAmpPCR 2720 (Applied Biosystems, CA, USA)^56^. The cDNAs were amplified with primers specific for mTOR (5′-GTCATCCAACGGGAATGCA-3′/5′- TGATCGGTTACCGTGATCAAAA-3′), GAPDH (internal control): (5′-CAGAACATCATCCTGCCTCT- 3′/ 5′-GCTTGACAAAGTG GTCGTTGAG-3′)^13^.

### Chromatin immunoprecipitation (ChIP)

ChIP assays were executed with the help of a ChIP assay kit (Millipore, Darmstadt, Germany) following manufacturer’s protocol^54^. PCR assay for identification of RNA pol II (Santa Cruz, CA, USA) (Cat No. sc-47701), NFκB and P300 binding regions on *mTOR* promoter (TATA-box region as well as INR region in case of RNA pol II) was performed using different primer sets.

For RNA pol II on TATA box:

5′GACCCATACAACCCTTTTTCC3′/5′GGAACCACCGGACATTCTCT3′;

For RNA pol IIon INR region:

5′CGCTTCCCCCTTCCTTTTTC3′/5′CAGCCTCTGGTGTTAATGAGAGC3′;

For NFκB and P300 binding on mTOR region:

5′AGACCTTGGGTCGGCTCAGT3′/5′ACCGGAAACCTCGCCCAATC3′

Extracted DNA (2 µl) was used for 45 cycles of amplification in 20 µl of reaction mixture under the following conditions: 95 °C for 30 s, 56 °C for 30 s, and 72 °C for 60 s^56^. The PCR products were subjected to electrophoresis using 2% agarose gel^56^.

### Plasmids, siRNA and transfections

The expression constructs Myr-Akt/DN-Akt (a kind gift from Professor Nissim Hay of University of Illinois at Chicago) and control pcDNA3.0 vectors (2 µg/million cells) were introduced into spheroids using lipofectamine-2000 (Invitrogen, Carlsbad) in accordance with manufacturer’s protocol. Isolation of stably expressing clones were carried out by limiting dilution followed by selection with G418 sulfate (1 mg/ml; Cellgro)and G418 resistant cells were next cloned and screened by immunoflourescence or western blotting with specific antibodies^20^. Endogenous silencing of target genes was achieved by transfecting cells with 300pmol of E-cadherin-/control-siRNA using lipofectamine-2000 separately for 12 h. The mRNA and protein levels were analyzed by RT-PCR and Western blotting, respectively^57^.

### Cycloheximide Assay

Following the method used by Saha *et al*.^4^, spheroids were maintained in DMEM/F12 supplemented with 5 μg/mL bovine insulin (Sigma-Aldrich), 20 ng/mL basic fibroblast growth factor, 20 ng/mL recombinant epidermal growth factor, 0.4% bovine serum albumin (BSA)and B27 supplement (BD Biosciences, San Jose, CA, USA). After 24 h of incubation 50 µg/ml of cycloheximide (Sigma) was added to the medium and the decay in the steady state level of a specific target protein was monitored by immunoblotting at indicated time points^58^.

### Oncomine data analyses

Outlier analysis of gene expression data for CD44 or CD133 were performed in the publicly available Oncomine database (Compendia Bioscience, Ann Arbor, MI, USA) using primary filters for analyses type of cancer vs. normal analylsis, cancer type as lung cancer, sample filter to use clinical specimens and data set filters to use mRNA data sets. For analysing stage wise mRNA expression, an additional filter was used in the sample filter i.e pathology subtype-stage.

### KM plotter survival analyses

Kaplan–Meier analysis was performed using publicly available database (http://kmplot.com/analysis/) with the following options: Affy id: 207163_s_at; splitting patients by ‘Autoselect best cutcoff’; survival: first progression (FP) or overall survival (OS); probe set option: only Jetset best probe set and restricting analyses to subtype: adenocarcinoma.

### Statistical analysis

Values are shown as standard error of mean, except otherwise indicated. Data were analyzed and, appropriate, significance (p < 0.05) of the differences between mean values was determined by a Student’s t test^13,59,60^.

## Supplementary information

Supplementary Figures
