# Supplementary material for: Aspirin enhances cisplatin sensitivity of resistant non-small cell lung carcinoma stem-like cells by targeting mTOR-Akt axis to repress migration
Source: Sci Rep. 2019 Nov 15;9:16913. doi: 10.1038/s41598-019-53134-0 (PMC6858356; doi:10.1038/s41598-019-53134-0)
Supplement: Supplementary file 1 — Supplementary Figures [file 41598_2019_53134_MOESM1_ESM.pdf]

Research Article

Aspirin enhances cisplatin sensitivity of resistant non-small cell lung carcinoma stem-like cells by targeting mTOR-Akt axis to repress migration

Poulami Khan, Apoorva Bhattacharya, Debomita Sengupta, Shruti Banerjee, Arghya Adhikary[#] and Tanya Das*

Division of Molecular Medicine, Bose Institute, P-1/12, CIT Scheme VII M, Kolkata 700054, India

[#]Present address: Centre for Research in Nanoscience and Nanotechnology, University of Calcutta, JD-2, Sector III, Salt Lake, Kolkata 700098, West Bengal, India

Running Title: Aspirin ensures anti-migratory effect of cisplatin

***To whom correspondence should be addressed: Prof. Tanya Das, Division of Molecular Medicine, Bose Institute, P-1/12, CIT Scheme VII M, Kolkata 700 054, India.**

Tel: +91-33-2569-3257; Fax: +91-33-2355-3886; E-mail: tanya@jcbose.ac.in

Key Words: Akt, aspirin, cancer stem cells, cisplatin, integrin, mTOR, migration

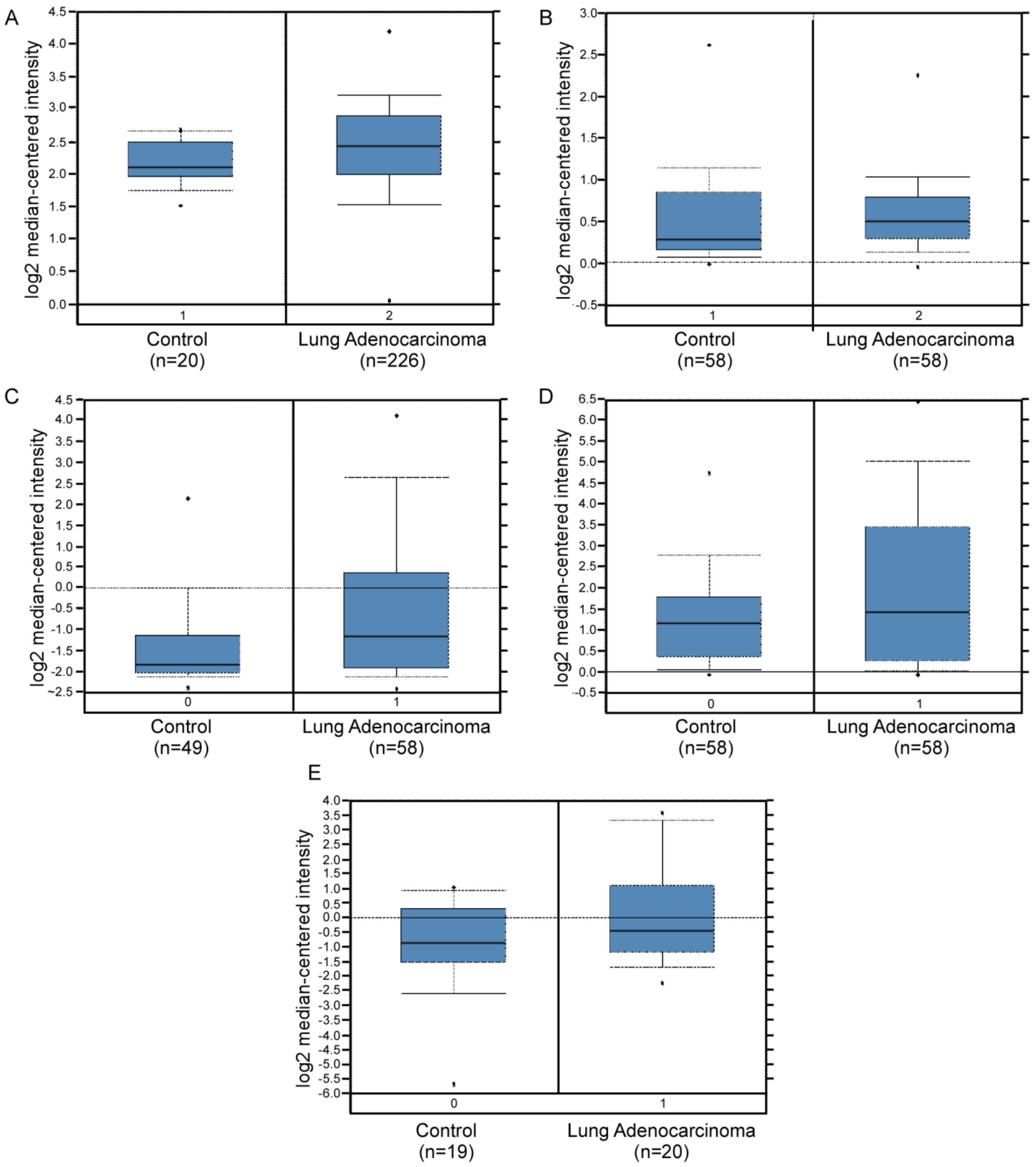

Figure S1: ***In silico*** **analysis demonstrating expressions of human lung cancer stem cell markers CD44 and CD133 in clinical specimens.** (A) Box whiskar plots from Okayama Lung dataset (n=246) and (B) Selamat Lung (n=116) dataset demonstrating CD44 mRNA expression in control vs. lung adenocarcinoma. (C) Box whiskar plots from Landi Lung (n=107), (D) Selamat Lung (n=116) and (E) Stearman Lung (n=39) dataset representing CD133 mRNA expression in control vs. lung adenocarcinoma.

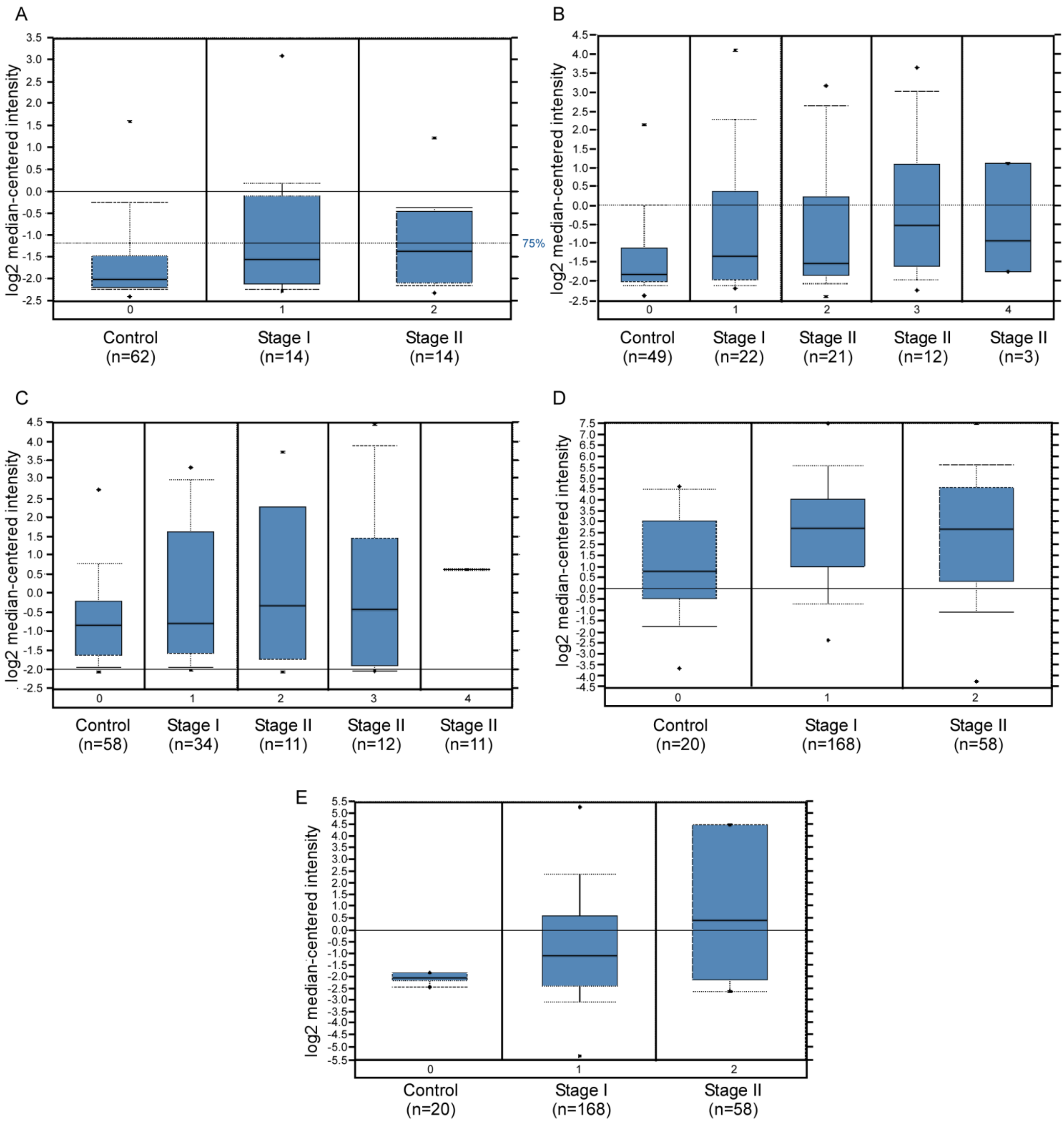

Figure S2: Stage-wise involvement of CD133 in lung adenocarcinoma. (A) Box whiskar plots from Zhu Lung (n=90), (B) Landi Lung (n=107), (C) Selamat Lung (n=116), (D) Okayama Lung (n=246), and (E) TCGA Lung (n=246) dataset representing stage-wise CD133 mRNA expression in lung adenocarcinoma patients.
